# Association of Titin Polymorphisms with the Progression of Oral Squamous Cell Carcinoma and Its Clinicopathological Characteristics

**DOI:** 10.3390/ijms25189878

**Published:** 2024-09-12

**Authors:** Ching-Hui Hsu, Mu-Kuan Chen, Yu-Sheng Lo, Hsin-Yu Ho, Chia-Chieh Lin, Yi-Ching Chuang, Ming-Ju Hsieh, Ming-Chih Chou

**Affiliations:** 1 Department of Otorhinolaryngology, Head and Neck Surgery, Changhua Christian Hospital, Changhua 50006, Taiwan; 2 Institute of Medicine, Chung Shan Medical University, Taichung 40201, Taiwan; 3 Department of Post-Baccalaureate Medicine, College of Medicine, National Chung Hsing University, Taichung 40201, Taiwan; 4 Graduate Institute of Clinical Medicine, College of Medicine, National Chung Hsing University, Taichung 40201, Taiwan; 5 Oral Cancer Research Center, Changhua Christian Hospital, Changhua 40201, Taiwan183581@cch.org.tw (H.-Y.H.);; 6 Graduate Institute of Biomedical Sciences, China Medical University, Taichung 406040, Taiwan; 7 Division of Thoracic Surgery, Department of Surgery, Chung Shan Medical University Hospital, Taichung 40201, Taiwan

**Keywords:** *TTN*, oral cancer, polymorphism, cigarette smoking

## Abstract

This study examined the correlation of titin (*TTN*) polymorphisms with the sensitivity of oral squamous cell cancer (OSCC) and clinical characteristics. Six *TTN* SNPs, including rs10497520, rs12463674, rs12465459, rs2042996, rs2244492, and rs2303838, were evaluated in 322 control groups and 606 patients with oral cancer. We then investigated whether the SNP genotypes rs10497520 had associations with clinical pathological categories. Our data showed that the TC + CC genotype of rs10497520 was associated with moderate/poor tumor cell differentiation. The carriers of *TTN* rs10497520 polymorphic variant “TC + CC” in OSCC patients with cigarette smoking were linked with poor tumor differentiation (*p* = 0.008). Our results suggest that the *TTN* SNP rs10497520 is a possible genetic marker for oral cancer patients in the cigarette-smoking population. The *TTN* rs10497520 polymorphisms may be essential biomarkers to predict the onset and prognosis of oral cancer disease.

## 1. Introduction

Oral squamous cell carcinoma (OSCC), a subtype of the head and neck squamous carcinoma that occurs in the oral cavity, is one of the fastest growing public health problems in the world, affecting millions of people annually; rates are increasing at an alarming rate [1]. OSCC is the eighth most common cancer worldwide [2]. “Oral cancer” primarily refers to cancerous lesions that start in the oral cavity, such as the tongue, the floor of the mouth, the palate, the gingiva, the vermilion border of the lip, and the buccal mucosa [3]. OSCC is also the leading cause of cancer-related death, and is most common in men, middle-aged, and older adults [4]. OSCC is usually caused by a combination of genetic and environmental factors, including smoking, alcohol consumption, and betel nut consumption [5]. Early diagnosis and prompt treatment are crucial for the successful treatment of oral squamous cell carcinoma; preventive strategies such as avoiding smoking and alcohol consumption are some of the keys to reducing the burden of oral cancer. However, the development of OSCC is mainly influenced by environmental variables and the accumulation of genetic abnormalities [6]. Research has linked single nucleotide polymorphisms (SNP) to various types of cancer and found that genetic polymorphisms influence cancer susceptibility induced by changes in gene regulation [7]. The analysis of putative functional SNPs in genes can significantly improve human health by guiding the selection of treatments [8].

Titin, also known as the *TTN* gene, is a large molecule that plays a vital role in muscle contraction, particularly in striated muscles such as skeletal and cardiac muscles [9]. Titin comprises multiple repeating structural motifs and domains that act as a molecular spring; sarcomeres are the basic unit of muscle contraction. It spans from the Z disk to the M line within the sarcomere, providing structural support and contributing to the passive elasticity of the muscle [10,11]. In addition to controlling many of the mechanical properties of sarcomeres during rest and contraction, titin plays a significant role in the formation and transmission of signals within sarcomeres [9]. Mutations in the *TTN* gene can lead to various muscle-related disorders. Mutations responsible for disease-associated variants of *TTN* include nonsense, missense, and truncated mutations, insertion/deletion mutations, and splice mutations [12]. These may include certain types of muscular dystrophy and cardiomyopathies [11,13]. The specific effects of *TTN* gene mutations can vary, but often result in abnormalities in muscle structure or function. A novel genetic mechanism underlying the response to exercise-induced muscle damage (EIMD) in humans can be discovered by examining two *TTN* SNPs associated with exercise-induced muscle damage in vivo (rs3731749) and with changes in muscle stem cell migration after damage in vitro (rs1001238) [14]. The *TTN* SNPs were associated with the status of the estrogen or progesterone receptor in breast cancer, including rs12463674 for low histological grade and rs2244492 for low hormone receptor status [15]. Calcium and hormones play a crucial role in muscle contraction. However, no research has been conducted on the connection between *TTN* SNPs and oral cancer. Here, we evaluate the impact of *TTN* SNPs (rs10497520, rs12463674, rs12465459, rs2042996, rs2244492, rs2303838) on the risk of OSCC.

## 2. Results

### 2.1. Clinicopathological Characteristics of OSCC Patients

Table 1 provides an overview of the clinicopathological and demographic characteristics of OSCC patients. There was no statistically significant variation in age distribution between the normal control group and patients with oral cancer (*p* = 0.06). Regarding carcinogenic substances and lifestyle choices, 66.3% of the patient group chewed betel quid, compared to only 4% in the control group (*p* < 0.001); 79.4% of the patient group smoked cigarettes, compared to 7.1% in the control group (*p* < 0.001); 34% of the patient group drank alcohol, compared to 2.8% in the control group (*p* < 0.001). These findings indicated a strong correlation between alcohol consumption, smoking, chewing of betel quid, and OSCC (Table 1). According to the clinicopathological distributions, 44.2% and 55.8% of the patient group cases were classified as early stage (stages I and II) and late stage (stages III and IV). Patients with tumors less than 4 cm (T1 + T2) made up 58.6% of the patient group in terms of tumor size (tumor T status), while 41.4% of the patients had more than 4 cm of tumors (T3 + T4). Regarding regional lymph node metastases, approximately two-thirds of the patients (67.7%) were classified as N0, while the remaining 32.3% had lymphatic metastases (N1 + N2 + N3). Patients with M0 had a metastatic status of 96.7%, while those with M1 had a distant metastasis rate of 3.3%. According to the histological studies, 86% of the patients had moderate or poor cell differentiation (Table 1).

### 2.2. Association of TTN Genetic Variants with the Incidence of OSCC

Using logistic regression models, we computed odds ratios (ORs) to investigate the relationship between the five independent SNPs of *TTN* and the incidence of oral cancer. The risk of oral cancer was not significantly correlated with these SNPs in either the cancer or control groups. After correcting odds ratios (AOR) with a 95% confidence interval (CI) for risk variables, such as chewing betel nut, drinking alcohol, and using tobacco, similar findings were observed between *TTN* SNPs and oral cancer patients (Table 2).

### 2.3. Clinical Status and TTN rs10497520 Genotype Frequencies in the OSCC Group

Subsequently, we examined the polymorphic associations between genotypes and the clinical pathological characteristics of OSCC patients to determine if the SNP rs10497520 exhibited any connection with variable categories (Table 3). According to statistical findings, individuals with the TC + CC allele of rs10497520 were more likely than those with the TT genotype to experience cell differentiation (*p* = 0.008, OR = 2.012, 95% CI = 1.201–3.370) (Table 3). However, no correlation was observed between the rs10497520 genotypes and other characteristics, including clinical stage, tumor size, lymph node metastasis, or distant metastases. Table 4 presents the results of an investigation into possible relationships between the *TTN* rs10497520 polymorphism and the risk of OSCC and alcohol consumption, the chewing of betel nuts, and smoking. According to our research, there was no link between the clinical stage, tumor size, lymph node metastasis, distant metastasis, or cell differentiation of participants in the alcohol-drinking and betel-nut-chewing group in the *TTN* SNP rs10497520 subgroup. However, the cigarette smoking group demonstrated that patients with the TT allele were more likely than patients with the TC + CC genotype to have poor cell differentiation. These findings were statistically significant (*p* = 0.008).

## 3. Discussion

Titin (*TTN*) is more widely recognized for its structural and elastic functions in the muscle contractile machinery [12]. *TTN* is one of the most commonly mutated genes in solid tumors, such as gastric cancer tumors [13], and other tumor types such as lung cancer, breast cancer, and colon cancer [14]. Although the role of *TTN* in oral cancer is limited, in this study, our aim was to investigate the potential genetic polymorphisms of *TTN* in Taiwanese patients with oral cancer. We examined the relationship between oral cancer susceptibility and *TTN* genotypic frequencies, including rs12463674, rs12465459, rs2042996, rs2244492, and rs2303838. Our research did not reveal statistically significant differences between controls and oral cancer patients, demonstrating a limited carcinogenic effect and limited disease sensitivity of *TTN* polymorphisms to oral cancer.

Our further analysis found that patients who carry at least one minor allele of rs10497520 (TC and CC; OR, 2.012; 95% CI, 1.201–3.370; *p* = 0.008) were more prone to developing poor tumor differentiation (Table 3). Given that smoking, eating betel nuts, and drinking alcohol are known risk factors for OSCC [15], we calculated and compared the different genotypes of the patients. Our research showed that patients with the TT allele who smoked cigarettes were more likely than patients with the TC + CC allele to have poor cell differentiation (*p* = 0.008). These results are similar with the research obtained by Göhler et al., where it was demonstrated that six *TTN* SNPs are associated with a higher risk of breast cancer, aggressive tumor features, and a lower chance of survival [16]. The risk of BC was correlated with homozygosity for the minor allele of rs10497520: C > T (OR = 1.96 [95% CI = 1.18–3.26], *p* = 0.01) [16]. Previous studies have also found that overexpression of titin in colorectal cancer (CRC) cells promotes proliferation and metastasis, and that a lower survival rate was noticeably correlated with a higher titin expression level in CRC tumors [17]. In contrast to our findings, the study from Fernandez-Moya et al. presented different patterns related to *TTN* SNPs and cancer [18]. In Chilean women with negative BRCA1/2 with a strong family history of BC or early-onset nonfamilial BC, the rs10497520-T allele, homozygosity of T/T, or those that harbored the T allele (C/T + T/T) exhibited a protective effect. This evidence demonstrates that the *TTN* SNPs may serve as positive or negative biomarkers in different types of tumor cells.

However, this study has several limitations. Patient samples were categorized by location into the tongue, buccal mucosa, and other sites (including gums, lips, and palate), with 220 samples from the tongue, 210 from the buccal mucosa, and fewer than 60 from other sites. While we conducted a detailed analysis by location, the small sample size led to biased results. Therefore, we plan to collect more samples to build a larger, more robust dataset for future analyses. In addition, functional research is required to determine the biological significance of these variations.

## 4. Materials and Methods

### 4.1. Patients and Specimens

A total of 928 samples were collected from patients at Changhua Christian Hospital in this study; each patient’s written informed consent was obtained before participation in the study started. Between 2014 and 2023, Changhua Christian Hospital enrolled 606 patients with OSCC and 322 patients without cancer as a control group. A protocol for this study was approved by the Institutional Review Board (IRB) of Changhua Christian Hospital (CCH) under number 130616. All participants in the study lived in Han Chinese communities and there was no geographical difference between them. A statistical analysis of medical records confirmed the age and habits of the participants (such as chewing betel nuts, smoking, and drinking alcohol). Additionally, AJCC No. 8 also discussed the evaluation of the clinical stage, the stage of tumor/lymph node/metastasis (TNM), and the degree of cell differentiation in cancer cells [19]. The researchers collected venous blood samples and stored them in K3–ethylene diamine tetraacetic acid (EDTA) (Merck Millipore, Burlington, MA, USA) test tubes. Blood samples were cryogenically centrifuged and stored in −80 °C laboratory refrigerator (PHC Corporation, Biomedical Division, Gunma, Japan) for analysis.

### 4.2. Functional TTN SNP Selection

We evaluated the likelihood of mutations, conservation, and the recombination rate (RR) of genes and SNPs associated with survival and tumor characteristics to gain insights into their functional consequences. Previous research on functional variants of breast cancer driver genes identified six polymorphisms, including five missense mutations (rs10497520, rs12463674, rs2042996, rs2244492, and rs2303838) and one promoter region mutation (rs12465459) [15,16]. The selection of SNPs was based on the following criteria: (1) minor allele frequencies (MAF) greater than 10%; (2) location within the coding regions (non-synonymous SNPs), promoter regions, 5′ and 3′ untranslated regions (UTR); and (3) Haploview was used to select SNPs based on linkage disequilibrium (LD; r^2^ ≥ 0.80) to minimize the number of SNPs to be genotyped.

### 4.3. Genomic DNA Extraction

Patient blood samples were collected into sterile tubes containing EDTA during hospitalization, centrifuged, and kept at −80 °C for later analysis. A previous method of extracting genomic DNA from peripheral blood leukocytes (Qiagen, Valencia, CA, USA) was used to extract DNA from peripheral blood leukocytes [20]. The extracts were dissolved in TE buffer (1 mM ethylenediaminetetraacetic acid and 10 mM tris aminomethane; pH 7.8) (Merck Millipore, Burlington, MA, USA). The extracts were dissolved. The optical density was measured at 260 nm. The extracts were then stored at −20 °C.

### 4.4. Real-Time PCR

The three *TTN* SNPs rs10497520 (T/C), rs12463674 (A/G), rs12465459 (T/C), rs2042996 (A/G), rs2244492 (C/T), and rs2303838 (T/C) obtained through the above analysis were included in the analysis model. These genotyping assays were ordered from Applied Biosystems with TaqMan-minor groove binder (MGB) moiety genotyping assay mix. The probe IDs for the TaqMan-SNP Genotyping Assay Data Sheet were C_1958912_10 (rs10497520), C_2071730_10 (rs12463674), C_3144639_10 (rs12465459), C_2071743_10 (rs2042996), C_16279790_10 (rs2244492), and C_2071738_30 (rs2303838); all probes were stored at −20 °C. As part of the quantitative real-time PCR analysis, the polymorphisms rs10497520 (T/C), rs12463674 (A/G), rs12465459 (T/C), rs2042996 (A/G), rs2244492 (C/T), and rs2303838 (T/C) were determined using the real-time PCR system ABI StepOne (Applied Biosystems, Foster City, CA, USA); StepOne Software v2.3 (Applied Biosystems, Foster City, CA, USA) was used to analyze the results. We conducted real-time PCR tests on the polymorphisms of the *TTN* gene, as previously described [21]. To create each reaction, 2.5 µL of TaqMan Genotyping Master Mix, 0.125 µL of TaqMan probe mix and 30 ng genomic DNA were combined, resulting in a final volume of 5 µL. Following an initial denaturation step at 95 °C for 10 min, 40 amplification cycles were conducted at 95 °C for 15 s and 60 °C for 1 min.

### 4.5. Statistical Analysis

A similar analysis was performed in a previous study using IBM SPSS Statistics v22.0 (IBM, Armonk, NY, USA) to analyze the data collected from the clinical characteristics data [11]. The demographic significance of OSCC compared to noncancer controls was assessed using Mann–Whitney U validation. In the OSCC case versus the noncancer control group, the odds ratio distribution (OR) of the *TTN* SNP was calculated using logistic regression. We used logistic regression to evaluate the SNP after correction for chewing, smoking and alcohol consumption of betel quid. In contrast, multiple regression was used to calculate adjusted odds ratios (AOR) with 95% confidence intervals (CI) for the *TTN* SNP distribution. However, multiple logistic regression models explored the relationship between genotype frequency, OSCC risk, clinical status, and potential confounding variables. The threshold definitions for differences or associations set *p* values at <0.05.

## 5. Conclusions

In summary, our findings demonstrate an interaction relationship between cell differentiation and *TTN* rs10497520 in OSCC patients. Moreover, *TTN* rs10497520 was highlighted as important in the tobacco-smoking group, particularly in relation to the poor progression of OSCC. Nevertheless, more studies are necessary on various ethnicities to evaluate the functional impact of rs10497520 (*TTN*) on OSCC tumorigenesis.

## Figures and Tables

**Table 1:** Demographical characteristics and clinical parameters were distributed in 322 controls and 606 cases with OSCC.

Variable	Control (N = 322)	Patients (N = 606)	*p* Value
**Age (yrs.)**	53.20 ± 8.11	53.79 ± 8.76	
>54	168 (52.2%)	274 (45.2%)	*p* = 0.0619
≤54	154 (47.8%)	332 (54.8%)	
**Betel nut chewing**			
No	309 (96.0%)	204 (33.7%)	*p* < 0.0001 *
Yes	13 (4.0%)	402 (66.3%)	
**Cigarette smoking**			
No	299 (92.9%)	125 (20.6%)	*p* < 0.0001 *
Yes	23 (7.1%)	481 (79.4%)	
**Alcohol drinking**			
No	313 (97.2%)	400 (66.0%)	*p* < 0.0001 *
Yes	9 (2.8%)	206 (34.0%)	
**Stage**			
I + II		268 (44.2%)	
III + IV		338 (55.8%)	
**Tumor T status**			
T1 + T2		355 (58.6%)	
T3 + T4		251 (41.4%)	
**Lymph node status**			
N0		410 (67.7%)	
N1 + N2 + N3		196 (32.3%)	
**Metastasis**			
M0		586 (96.7%)	
M1		20 (3.3%)	
**Cell differentiation**			
Well differentiated		85 (14.0%)	
Moderately or poorly differentiated		521 (86.0%)	

N: number. * *p* value < 0.05 as statistically significant.

**Table 2:** The distribution of genotype frequencies in *TTN* SNPs in cases of the OSCC group.

Variable	Control (N = 322)	Patients (N = 606)	OR ^a^ (95% CI)	AOR ^b^ (95% CI)
**rs10497520**				
TT	212 (65.8%)	369 (60.9%)	1.000	1.000
TC	93 (28.9%)	214 (35.3%)	1.322 (0.983–1.778)	1.210 (0.793–1.844)
CC	17 (5.3%)	23 (3.8%)	0.777 (0.406–1.488)	0.859 (0.341–2.164)
TC + CC	110 (34.2%)	237 (39.1%)	1.238 (0.933–1.642)	1.156 (0.772–1.729)
**rs12463674**				
AA	297 (92.2%)	536 (88.4%)	1.000	1.000
AG	24 (7.5%)	66 (10.9%)	1.524 (0.935–2.483)	1.717 (0.892–3.302)
GG	1 (0.3%)	4 (0.7%)	2.216 (0.247–19.92)	3.518 (0.255–48.46)
AG + GG	25 (7.8%)	70 (11.6%)	1.551 (0.962–2.503)	1.785 (0.944–3.378)
**rs12465459**				
TT	274 (85.1%)	502 (82.8%)	1.000	1.000
TC	44 (13.7%)	99 (16.3%)	1.228 (0.836–1.804)	1.247 (0.729–2.133)
CC	4 (1.2%)	5 (0.8%)	0.682 (0.182–2.562)	1.109 (0.191–6.449)
TC + CC	48 (14.9%)	104 (17.2%)	1.183 (0.815–1.716)	1.236 (0.736–2.078)
**rs2042996**				
AA	163 (50.6%)	303 (50.0%)	1.000	1.000
AG	131 (40.7%)	240 (39.6%)	0.986 (0.741–1.311)	1.011 (0.671–1.525)
GG	28 (8.7%)	63 (10.4%)	1.210 (0.746–1.964)	0.841 (0.416–1.702)
AG + GG	159 (49.4%)	303 (50.0%)	1.025 (0.782–1.343)	0.978 (0.662–1.444)
**rs2244492**				
CC	230 (71.4%)	410 (67.7%)	1.000	1.000
CT	80 (24.8%)	180 (29.7%)	1.262 (0.927–1.719)	1.252 (0.805–1.948)
TT	12 (3.7%)	16 (2.6%)	0.748 (0.348–1.608)	1.074 (0.374–3.086)
CT + TT	92 (28.6%)	196 (32.3%)	1.195 (0.889–1.606)	1.229 (0.805–1.876)
**rs2303838**				
TT	130 (40.4%)	225 (37.1%)	1.000	1.000
TC	137 (42.5%)	279 (46.0%)	1.177 (0.874–1.584)	1.071 (0.698–1.643)
CC	55 (17.1%)	102 (16.8%)	1.072 (0.724–1.587)	0.960 (0.546–1.688)
TC + CC	192 (59.6%)	381 (62.9%)	1.147 (0.869–1.512)	1.038 (0.697–1.547)

N: number. ^a^ The odds ratio (OR) with its 95% confidence intervals was estimated by logistic regression models. ^b^ The adjusted odds ratio (AOR) with its 95% confidence intervals was estimated by multiple logistic regression models after controlling for betel nut chewing, alcohol, and tobacco consumption.

**Table 3:** Clinical statuses and *TTN* rs10497520 genotype frequencies in cases of the OSCC group.

Variable	*TTN* (rs10497520)			
	TT (%) (N = 369)	TC + CC (%) (N = 237)	OR ^a^ (95% CI)	*p* Value
**Clinical stage**				
Stage I/II	172 (46.6%)	96 (40.5%)	1.000	*p* = 0.140
Stage III/IV	197 (53.4%)	141 (59.5%)	1.282 (0.922–1.784)	
**Tumor size**				
T1 + T2	220 (59.6%)	135 (57.0%)	1.000	*p* = 0.517
T3 + T4	149 (40.4%)	102 (43.0%)	1.116 (0.801–1.553)	
**Lymph node metastasis**				
No	252 (68.3%)	158 (66.7%)	1.000	*p* = 0.676
Yes	117 (31.7%)	79 (33.3%)	1.077 (0.761–1.525)	
**Distant metastasis**				
No	355 (96.2%)	231 (97.5%)	1.000	*p* = 0.399
Yes	14 (3.8%)	6 (2.5%)	0.659 (0.250–1.738)	
**Cell differentiation**				
Well	63 (17.1%)	22 (9.3%)	1.000	*p* = 0.008 *
Moderate/poor	306 (82.9%)	215 (90.7%)	2.012 (1.201–3.370)	

N: number. **^a^** The logistic regression model estimated the odds ratio (OR) with their 95% confidence intervals. * *p* value < 0.05 as statistically significant.

**Table 4:** Clinical statuses and *TTN* rs10497520 genotype frequencies in cases of the OSCC group among betel nut chewing, cigarette smoking, and alcohol drinking.

Variable	*TTN* (rs10497520)								
	Alcohol Drinking (N = 206)			Betel Nut Chewing (N = 402)			Cigarette Smoking (N = 481)		
	TT (%) (N = 122)	TC + CC (%) (N = 84)	*p* Value	TT (%) (N = 249)	TC + CC (%) (N = 153)	*p* Value	TT (%) (N = 294)	TC + CC (%) (N = 187)	*p* Value
**Clinical stage**									
Stage I/II	54 (44.3%)	35 (41.7%)	*p* = 0.712	121 (48.6%)	63 (41.2%)	*p* = 0.148	138 (46.9%)	82 (43.9%)	*p* = 0.508
Stage III/IV	68 (55.7%)	49 (58.3%)		128 (51.4%)	90 (58.8%)		156 (53.1%)	105 (56.1%)	
**Tumor size**									
T1 + T2	68 (55.7%)	49 (58.3%)	*p* = 0.712	146 (58.6%)	82 (53.6%)	*p* = 0.322	178 (60.5%)	109 (58.3%)	*p* = 0.623
T3 + T4	54 (44.3%)	35 (41.7%)		103 (41.4%)	71 (46.4%)		116 (39.5%)	78 (41.7%)	
**Lymph node metastasis**									
No	79 (64.8%)	55 (65.5%)	*p* = 0.915	178 (71.5%)	104 (68.0%)	*p* = 0.455	199 (67.7%)	131 (70.1%)	*p* = 0.586
Yes	43 (35.2%)	29 (34.5%)		71 (28.5%)	49 (32.0%)		95 (32.3%)	56 (29.9%)	
**Distant metastasis**									
No	115 (94.3%)	82 (97.6%)	*p* = 0.262	239 (96.0%)	150 (98.0%)	*p* = 0.268	238 (96.3%)	182 (97.3%)	*p* = 0.526
Yes	7 (5.7%)	2 (2.4%)		10 (4.0%)	3 (2.0%)		11 (3.7%)	5 (2.7%)	
**Cell differentiation**									
Well	18 (14.8%)	7 (8.3%)	*p* = 0.171	43 (17.3%)	16 (10.5%)	*p* = 0.063	53 (18.0%)	17 (9.1%)	*p* = 0.008 *
Moderate/poor	104 (85.2%)	77 (91.7%)		206 (82.7%)	137 (89.5%)		241 (82.0%)	170 (90.9%)	

N: number. * *p* value < 0.05 as statistically significant. OR (95% CI): 2.199 (1.231–3.930).

## Data Availability

The datasets generated for this study are available on request to the corresponding authors.
